# An open reduction and internal fixation of a Pipkin type 1 fracture: A case report

**DOI:** 10.1016/j.amsu.2022.104850

**Published:** 2022-11-08

**Authors:** Jafar Sallameh, Ebrahim Makhoul, Diala Nseir, Mawya Alkhayer, Ali Aboalchamlat

**Affiliations:** a Department of Orthopedic Surgery, Tishreen University Hospital, Latakia, Syria; b Cancer Research Center, Tishreen University Hospital, Latakia, Syria; c Department of Oncology, Tishreen University Hospital, Latakia, Syria; d Department of Rheumatology, Tishreen University Hospital, Latakia, Syria

## Abstract

• A rare case of a Pipkin fracture type 1.
• Emphasizing the importance of using clinical radiology precisely.
• Using ORIF procedure urgently has significant better outcomes.
• Protecting patients from femoral head necrosis and improving their mobility.

## 1 Introduction

Pipkins fractures are articular fractures of femoral head that are mainly caused by high-energy traumas like motor vehicle accidents (dashboard injury), sport injuries or falls from heights with an incidence of 2 cases per million [1,2]. These fractures are considered to be rarely encountered. However, this occurrence rate is increasing simultaneously with the higher incidence of road traffic accidents currently [1,2]. Since these fractures were described for the first time in 1869, multiple classification systems have emerged. However, Pipkin classification is the one to be most used till now. Pipkin classified these injuries into 4 types. Type 1 involves fracture inferior to the capitis femoris (the non-weight bearing part of femoral head) [1,2]. Radiology is the cornerstone to an appropriate assessment. Pelvic X-ray does not show the fragment in all cases and it depends on the size and location. Hence, the CT scan of pelvis is crucial to confirm the diagnosing. Femoral head fractures have been known to have bad functional results and high complication rates. The most considered complications are avascular necrosis (AVN) and post-traumatic arthritis of the hip joint. It is well documented that early reduction under anesthesia and adequate muscle relaxation, stabilization, and rigid fixation provide stable and congruent joints, nevertheless, reduce potential complication rate [3]. The best surgical approach on whether to fix or excise the femoral head fragment, however, remains controversial.

## 2 Methods

Our case report is compliant with the SCARE Guidelines 2020 [10].

## 3 Case presentation

**Fig. 1:**
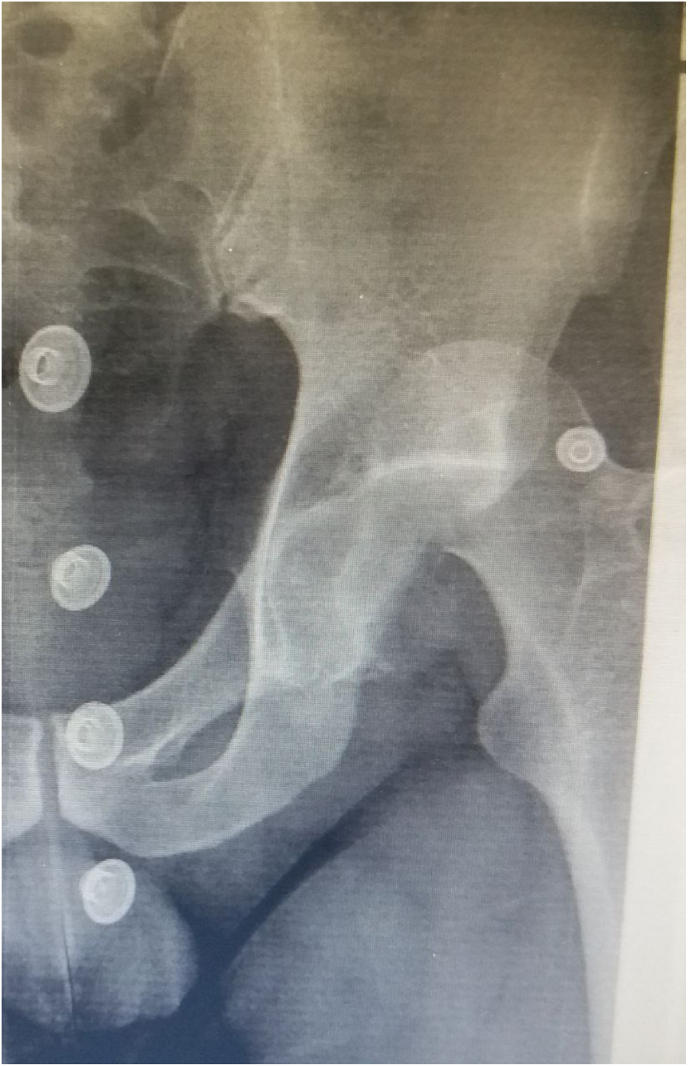
AP Preoperative X-ray of the pelvis shows the dislocation of the left hip joint A Computerized tomography (CT) scan of the hip showed a large femoral head fragment inferior to the fovea centralis (Fig. 2).

A 40 years old Caucasian male, who got exposed to a car accident (dashboard injury), was presented to the Emergency Department at Tishreen University Hospital. He had a Glasgow Coma Score (GCS) of 15/15. The patient sustained multiple lacerations on the left lower limb. His left lower limb was shortened, flexed at the hip, adducted, and internally rotated. A pelvic X-ray showed a posterior dislocation of the left hip associated with femoral head fracture (Fig. 1).

**Fig. 2:**
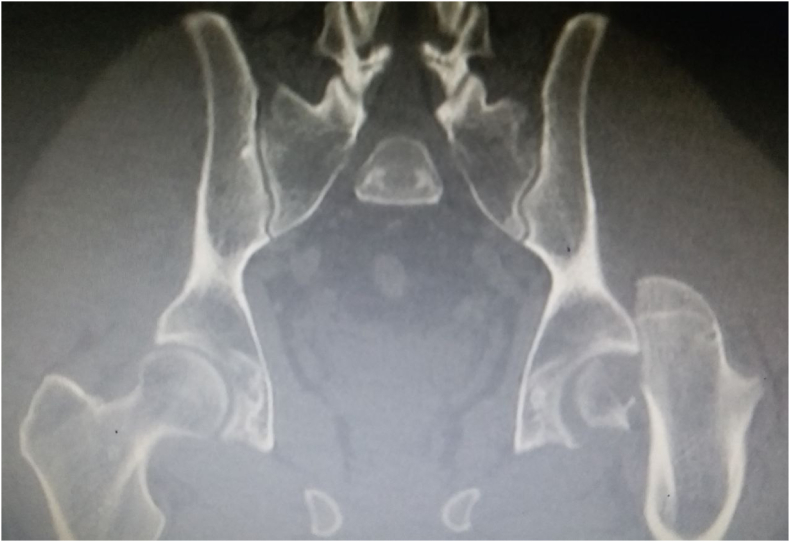
Preoperative CT scan of the pelvis shows the dislocation with the fracture of the femoral head, Pipkin fracture type 1.

**Fig. 3:**
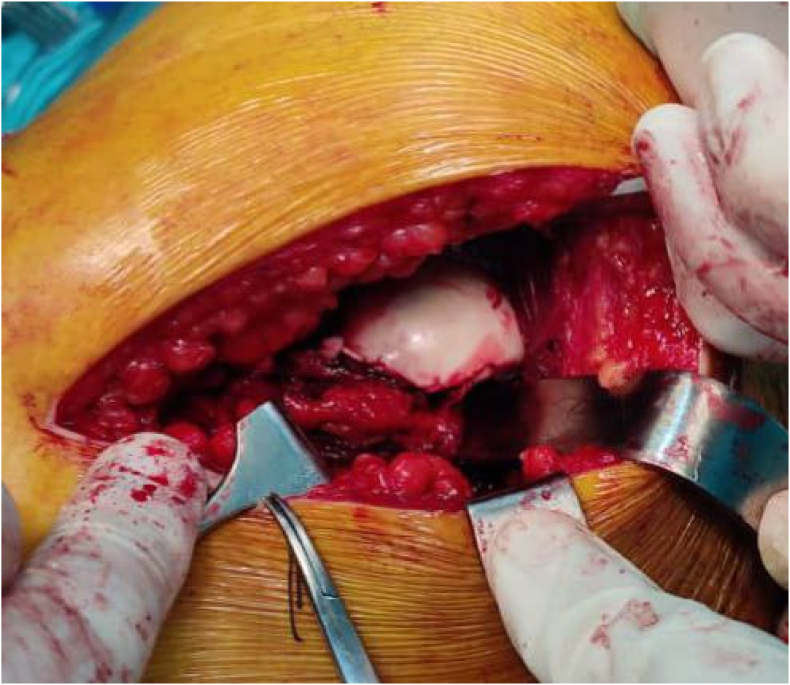
Intraoperative image showing the large femoral head fragment.

**Fig. 4:**
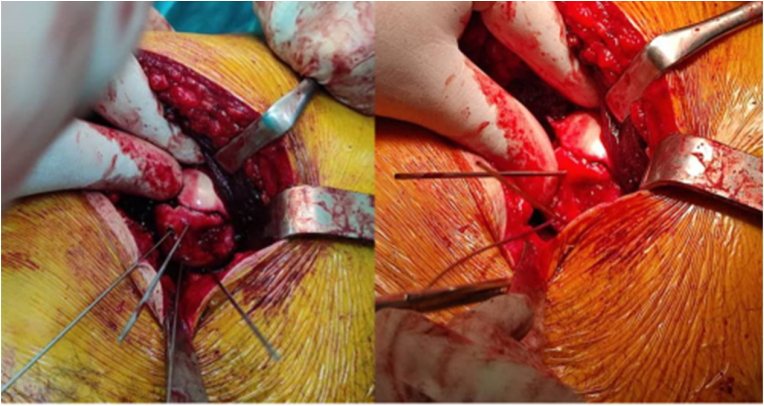
Reduced fragment fixed with Krshner wires.

All blood tests were within the normal range. A US (Ultra Sound) study was performed with negative results. A diagnosis of a posterior dislocation of the left hip with Pipkin type 1 was established. An urgent closed reduction under general anesthesia was achieved in 30 minutes of being presented to the emergency department and 2 hours after the injury. There was no associated neurovascular deficit before or after the reduction. Then, a decision of performing an open reduction and internal fixation ORIF for the fractured fragment was made (within 3 hours of admission) with an anterio-lateral approach. Intraoperatively, the fragment was found to be viable; hence, anatomical reduction was done followed by fixation of the fragment using three 2.7 mm subchondral headless cannulated screws (Herbert screws), and the capsule was closed with Vicryl 2 suture (Fig. 3, Fig. 4).

**Fig. 5:**
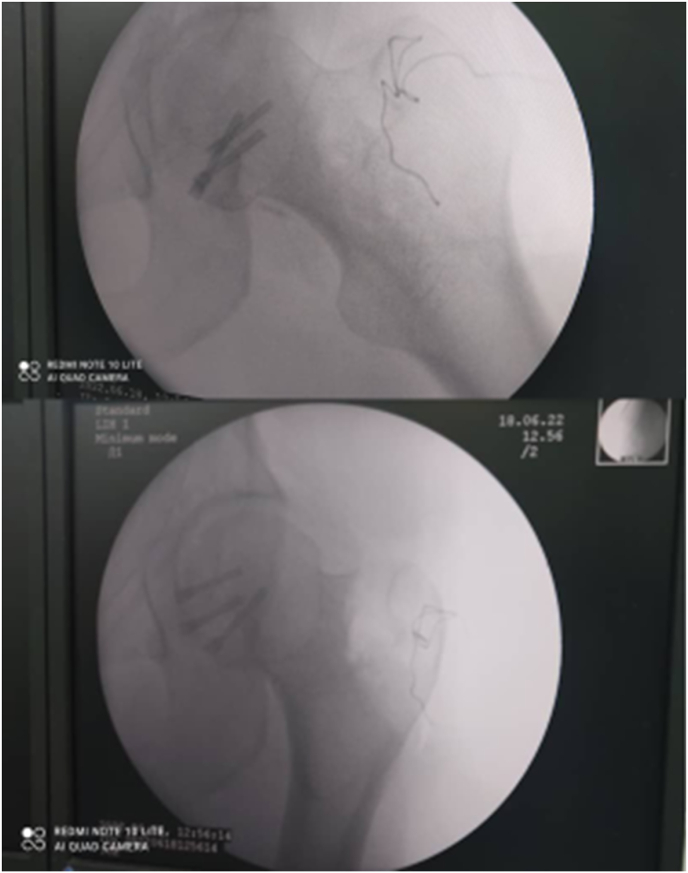
Postoperative pelvic X-ray showing screw fixation of the fractured fragment of the femoral head using three Herbert Screws. It is showing a good anatomical reduction.

The patient was discharged 3 days after the surgery with a good condition. The postoperative pelvic X-ray showed a good anatomical reduction (Fig. 5).

## 4 Discussion

Traumatic dislocations (12%) are associated with articular fractures that are considered rare. Fractures are often consequent to a traumatic posterior hip dislocation; less frequent are fractures caused by an anterior hip dislocation (11% of hip dislocations) or isolated femoral head articular fractures [4]. Since articular fractures are uncommon and localized in a deep joint, the final treatment results are challenging. Some complications after the treatment may develop due to the trauma: Heterotopic Ossifications (HO), Avascular Necrosis (AVN) and Osteoarthritis [5].

A very crucial factor in determining the positive outcome of treating the Pipkin fracture is obtaining a correct anatomical reduction of the fragments by an open surgery, especially, if the gap between the fragments were more than 2 mm [6]. Surgical procedures are recommended, in general, for types I and II Pipkin fractures with large fragments especially the ones related to the weight-bearing area of the head, nevertheless, types III and IV [7]. Moreover, one of the most important factors in treating hip joint dislocations, getting a satisfying outcome and avoiding femoral head necrosis is the time between the injury and reduction [8]. Hence, Pipkin fractures are rarely encountered, we need to diagnose these types of fractures quickly and efficiently. When our patient entered the emergency room, the X-Ray did not show the Pipkin fracture properly. However, CT-scan allowed us to see the fractured fragment precisely, make the right diagnosis, and acting urgently. Even though, the hip joint dislocation is a typical injury, we should always pay attention to the radiology materials carefully to observe such small and hidden fractures.

There are many different approaches to operate Pipkin fractures type 1 and the best one is a debatable issue [6,10]. When we first diagnosed the fracture, we decided to do an urgent open surgery because we knew the importance of investing time. The optimal timing of surgery is still controversial, but there are many studies suggest the importance of time as a crucial factor in determining the prognosis and decreasing complications [7]. The urgent surgery helped us to maintain the viability of the fragment, nevertheless, avoiding the femoral head necrosis. Through the surgery, the viability of the femoral head was confirmed by the bleeding from the fragment. The patient was discharged with very good results and he has not been having any complications until today (Three months after the surgery).

## 5 Conclusion

Through our case, we are describing a rare case of a Pipkin fracture type 1 and emphasizing the importance of using clinical radiology precisely to diagnose it. Nevertheless, we are referring to the crucial factor, the time, to operate such injuries to establish positive outcomes. Moreover, managing this type of cases using ORIF procedure urgently has significant better outcomes like protecting patients from femoral head necrosis and improving their mobility properly on the long range.

## Please state any conflicts of interest

No conflicts of interest.

## Please state any sources of funding for your research

No source of funding.

## Ethical approval

Informed consent was obtained from the patient regarding the report of their clinical scenario data in an anonymous way.

## Consent

Written informed consent was obtained from the patient for publication of this case report and accompanying images. A copy of the written consent is available for review by the Editorin-Chief of this journal on request.

## Author contribution

Jafar Sallameh and Ebrahim Makhoul: collected the data, drafted, discussed and edited the manuscript. Diala Nseir and Mawya Alkhayer: drafted and searched for similar cases in the literature. Ali Aboalchamlat: The guarantor and supervisor, critically revised the article and approved the final manuscript.

## Registration of research studies

1. Name of the registry:

An open reduction and internal fixation of a Pipkin type 1 fracture: A case report.

2. Unique Identifying number or registration ID: researchregistry8177.

3. Hyperlink to your specific registration (must be publicly accessible and will be checked): https://www.researchregistry.com/register-now#userresearchregistry/registerresearchdetails/62efe17ded6b770021d9cdad/

## Guarantor

Ali Aboalchamlat: Department of Orthopedic Surgery, Tishreen University Hospital, Latakia, Syria.
